# Further Evidence of Inadequate Quality in Lateral Flow Devices Commercially Offered for the Diagnosis of Rabies

**DOI:** 10.3390/tropicalmed5010013

**Published:** 2020-01-18

**Authors:** Antonia Klein, Anna Fahrion, Stefan Finke, Marina Eyngor, Shiri Novak, Boris Yakobson, Ernest Ngoepe, Baby Phahladira, Claude Sabeta, Paola De Benedictis, Morgane Gourlaouen, Lillian A. Orciari, Pamela A. Yager, Crystal M. Gigante, M. Kimberly Knowles, Christine Fehlner-Gardiner, Alexandre Servat, Florence Cliquet, Denise Marston, Lorraine M. McElhinney, Trudy Johnson, Anthony R. Fooks, Thomas Müller, Conrad M. Freuling

**Affiliations:** 1 Friedrich-Loeffler-Institut (FLI), Federal Research Institute for Animal Health, Institute of Molecular Virology and Cell Biology, 17493 Greifswald-Insel Riems, Germany; Antonia.Klein@fli.de (A.K.); Anna.Fahrion@fli.de (A.F.); Stefan.Finke@fli.de (S.F.); Thomas.Mueller@fli.de (T.M.); 2 Kimron Veterinary Institute (KVI), Veterinary Services and Animal Health, P.O. Box 12, Beit Dagan 50250, Israel; MarinaL@moag.gov.il (M.E.); shirin@moag.gov.il (S.N.); boris.yakobson@gmail.com (B.Y.); 3 Onderstepoort Veterinary Institute (OVI), Rabies Unit, Private Bag X05, Onderstepoort 0110, South Africa; NgoepeE@arc.agric.za (E.N.); PhahladiraB@arc.agric.za (B.P.); sabetac@arc.agric.za (C.S.); 4 Istituto Zooprofilattico Sperimentale delle Venezie, FAO Reference Centre for Rabies, Viale dell’Università, 10, 35020-Legnaro (PD), Italy; pdebenedictis@izsvenezie.it (P.D.B.); MGourlaouen@izsvenezie.it (M.G.); 5 Centers for Disease Control and Prevention (CDC), Poxvirus and Rabies Branch, Division of High-Consequence Pathogens and Pathology, National Center for Emerging and Zoonotic Infectious Diseases, Atlanta, GA 30329, USA; lao0@cdc.gov (L.A.O.); pay1@cdc.gov (P.A.Y.); lzu1@cdc.gov (C.M.G.); 6 Canadian Food Inspection Agency, Centre of Expertise for Rabies, Ottawa Laboratory Fallowfield, 3851 Fallowfield Road, Nepean, ON K2H 8P9, Canada; kim.knowles@canada.ca (M.K.K.); christine.fehlner-gardiner@canada.ca (C.F.-G.); 7 French Agency for Food, Environmental and Occupational Health and Safety (Anses), Laboratory for Rabies and Wildlife, Domaine de Pixérécourt, 54220 Malzéville CEDEX, France; Alexandre.servat@anses.fr (A.S.); florence.cliquet@anses.fr (F.C.); 8 Animal and Plant Health Agency (APHA), Weybridge, New Haw, Addlestone, Surrey KT15 3NB, UK; Denise.Marston@apha.gov.uk (D.M.); Lorraine.McElhinney@apha.gov.uk (L.M.M.); Trudy.Johnson@apha.gov.uk (T.J.); Tony.Fooks@apha.gov.uk (A.R.F.)

**Keywords:** rabies, diagnostics, lateral flow devices, validation

## Abstract

As a neglected zoonotic disease, rabies causes approximately 5.9 × 10^4^ human deaths annually, primarily affecting low- and middle-income countries in Asia and Africa. In those regions, insufficient surveillance is hampering adequate medical intervention and is driving the vicious cycle of neglect. Where resources to provide laboratory disease confirmation are limited, there is a need for user-friendly and low-cost reliable diagnostic tools that do not rely on specialized laboratory facilities. Lateral flow devices (LFD) offer an alternative to conventional diagnostic methods and may strengthen control efforts in low-resource settings. Five different commercially available LFDs were compared in a multi-centered study with respect to their diagnostic sensitivity and their agreement with standard rabies diagnostic techniques. Our evaluation was conducted by several international reference laboratories using a broad panel of samples. The overall sensitivities ranged from 0% up to 62%, depending on the LFD manufacturer, with substantial variation between the different laboratories. Samples with high antigen content and high relative viral load tended to test positive more often in the Anigen/Bionote test, the latter being the one with the best performance. Still, the overall unsatisfactory findings corroborate a previous study and indicate a persistent lack of appropriate test validation and quality control. At present, the tested kits are not suitable for in-field use for rabies diagnosis, especially not for suspect animals where human contact has been identified, as an incorrect negative diagnosis may result in human casualties. This study points out the discrepancy between the enormous need for such a diagnostic tool on the one hand, and on the other hand, a number of already existing tests that are not yet ready for use.

## 1. Introduction

Rabies is one of the most important yet neglected zoonotic diseases, causing approximately 5.9 × 10^4^ human deaths annually and primarily affecting low- and middle-income countries in Asia and Africa [1,2]. The deadly encephalitis is caused by different lyssaviruses, with the prototypical rabies virus (RABV) being their best-known representative and responsible for the vast majority of human cases [3]. All lyssaviruses belong to the Rhabdoviridae family in the order Mononegavirales [4]. The genome encodes for the five different viral proteins: nucleoprotein N, phosphoprotein P, matrix protein M, glycoprotein G, and the large polymerase L. All of these proteins are essential for virus replication and virus spread [5].

The virus is transmitted via infectious saliva, usually through bites or scratches of infected animals [3]. Dogs are the main source of human rabies, with approximately 99% of all human cases attributed to infections from rabid dogs [2]. Although resulting in a lethal disease, human rabies is completely preventable through vaccination of the dog reservoir to eliminate its source as well as through adequate and timely post-exposure prophylaxis (PEP), consisting of wound care and rabies vaccination in combination, when indicated, with rabies immunoglobulin [2]. At present, the United Against Rabies (UAR) consortium, comprising of the international organizations World Health Organization (WHO), World Organisation for Animal Health (OIE), Food and Agriculture Organization of the United Nations (FAO) and the Global Alliance for Rabies Control (GARC), have established a global strategic plan to reduce the burden of rabies, with the goal of reaching zero human deaths due to dog-mediated rabies by 2030 [6]. One of the pillars of this plan is to increase and harmonize rabies surveillance, ideally based on laboratory confirmation of clinically suspect animals to provide evidence-based guidance [7], i.e., both for bite case management and guidance for individual treatment as well as for general data aggregation to inform policymakers.

To this end, post mortem techniques recommended for the detection of the disease in animals [8] include the direct fluorescent antibody test (DFA), which was previously considered the gold standard and is still used as the reference method in most laboratories. The DFA is based on detecting viral antigen in brain impressions stained with fluorophore-conjugated antibodies by the use of fluorescence microscopy [9]. When biotin-conjugated antibodies are used in a direct rapid immunochemical test (DRIT), this technique offers the advantage that viral antigen can also be detected using light microscopy [10,11]. Various RT-PCRs have been established and validated to identify the presence of viral RNA [8]. Other tests, used mainly as confirmatory techniques, are based on virus isolation in cell culture, i.e., the rapid tissue culture infection test (RTCIT, [12]) or in mice [13]. Replacement of the latter by RTCIT is desirable for several reasons, including ethical considerations [8].

Rabies particularly occurs in regions of Africa and Asia that have limited access to healthcare or veterinary services [14], including adequate laboratory facilities with staff with high-level scientific expertise. Furthermore, often there are logistical constraints for sample shipment and storage, e.g., maintenance of a cold chain to ensure reliable results under tropical and subtropical conditions. Besides the fact that medical intervention, i.e., PEP, is not targeted without laboratory confirmation, lack of surveillance data is also one essential component that drives the vicious circle of neglect. Inaccurately low human and animal rabies case numbers belie the strong need for action [15,16].

Where laboratory-based disease confirmation is limited, there is a high demand for user-friendly and reliable low-cost diagnostic tools that rely neither on specific laboratory facilities nor on compound logistics. Lateral flow devices (LFDs), also known as rapid immunodiagnostic tests (RIDTs), immunodiagnostic assays or immunochromatographic strip tests, offer a promising alternative to conventional diagnostic methods and have the potential to strengthen prevention and control efforts in low-resource settings [17]. These LFDs are principally based on colloidal gold conjugated monoclonal antibodies that capture the antigen within a sample. The antigen–antibody complex thereupon binds to a second detection antibody that is fixed at the test zone “T“ on a nitrocellulose membrane, showing a colored line for a positive sample [18]. With their relative ease of test performance, which requires minimal staff training or scientific expertise, ambient storage temperature and minimal processing time, LFDs show great potential for in-field use for rabies diagnosis. Accordingly, the increased use of LFDs could help to overcome the limitations of disease detection and improve surveillance. However, prior to the adoption of such technology, the quality of these devices must be rigorously tested since life-saving PEP decisions may be contingent on results. False negative test results are unacceptable for a disease with case fatality of nearly 100%.

The Anigen/Bionote LFD is the only rabies test for which scientific evaluation has been published, showing promising results [19,20,21,22,23,24,25,26,27,28,29]. However, in a study from 2016, six different LFDs, including the Anigen/Bionote test, were evaluated with very unsatisfying results concerning their diagnostic reliability [30].

Since the completion of these previous studies, numerous LFDs marketed for rabies diagnosis have become available on the market. Based on current knowledge, none of those have passed any kind of national or international quality control or licensure procedures, such as in the United States [31] or Germany [32], indicating a lack of data regarding their sensitivity and specificity beyond the data from the test insert. We therefore assessed the diagnostic performance of these currently available LFDs in addition to Anigen/Bionote in relation to DFA and RT-qPCR using a comprehensively broad panel of samples within a multi-centered study. Furthermore, we determined the target of the diagnostic antibody used in the LFDs by assessing the reaction towards standardized samples consisting of transfected cells expressing only glycoprotein G, nucleoprotein N, matrix protein M, and phosphoprotein P, respectively.

## 2. Materials and Methods

### 2.1. Commercial LFD Test Kits for Rabies Diagnosis in Brain Material

To complement the analysis of a previous study [30], different commercial LFD test kits were selected based on availability and country of origin. The selected kits originated from five different countries and were purchased online. The acquired test kits were the Anigen Rapid Rabies Ag test kit (Bionote, Hwaseong-si, Korea; LOT NO: 1801DDO19), Intermedical Rapid Test Device Rabies Ag (Intermedical Diagnostics, Villaricca, Italy; LOT NO: Q007011701), LilliTest Rapid Rabies Ag test kit (Lillidale, Wimborne, United Kingdom; LOT NO: LRR041801), Elabscience rabies virus antibodies rapid test (Elabscience Biotechnology Inc., China/USA; LOT NO: AK0018JAN24057) and Span Biotech rapid rabies test (Span Biotec Ltd., Shenzhen, China; LOT NO: R04415970151). Each test kit consists of one test device, a cotton swab, a buffer solution tube or dropper bottle, and a small disposable pipette. All test kits require only little to no experience in laboratory work and can easily be performed after following the manufacturer’s user manuals; the Anigen kit provides a pictorial illustration of the most important steps and all kits provided illustrations of possible test results.

### 2.2. Participating Laboratories

Eight different OIE and FAO international reference laboratories for rabies were invited to participate voluntarily in this inter-laboratory comparison, from the following countries: Canada (Canadian Food Inspection Agency, CFIA), France (French Agency for Food, Environmental and Occupational Health & Safety, ANSES), Germany (Friedrich-Loeffler-Institut, FLI), Israel (Kimron Veterinary Institute, KVI), Italy (Istituto Zooprofilattico Sperimentale delle Venezie, FAO reference centre), South Africa (Onderstepoort Veterinary Institute, OVI), United Kingdom (Animal and Plant Health Agency, APHA), and USA (Centers for Disease Control and Prevention, CDC). Due to the limited numbers of kits, they were split amongst the participating laboratories, but could not be distributed equally to all. In general, the laboratories received at least ten tests per manufacturer and independently decided on the samples they included in the study.

### 2.3. Sensitivity Analyses

Diagnostic sensitivity of the commercial LFDs in comparison with DFA and RTqPCR results were investigated using a panel consisting of 132 different samples from already existing collections of frozen brain specimens. Each laboratory tested selected samples from their own collections, and no animals were used for this study.

All samples contained fresh or archived naturally infected brains or mouse brain homogenates generated from field strains after mouse inoculation. The panel comprised of 26 different genetic lineages of all major RABV genetic clusters (Arctic/Arctic-like, Asian, Cosmopolitan, New World), originating from 30 countries. Only RABV positive samples (N = 105) were taken into account for sensitivity analyses. All invalid test results were excluded from analysis, while faint lines were still considered as positive. The sensitivity was calculated using GraphPad Prism (Version 7), with confidence limits calculated according to Clopper and Pearson [33].

In addition to RABV variants, European bat lyssavirus type 1 (EBLV-1), European bat lyssavirus type 2 (EBLV-2), Bokeloh bat lyssavirus (BBLV), and Lleida bat lyssavirus (LLBV) infected mouse brains were included in this study at APHA.

For each sample, the DFA was performed according to the standard operating procedures of the respective laboratory, with results being quantified using a four-plus scoring system. Additionally, samples were subjected to real-time RT-PCR for confirmation and to determine the relative viral load with different RT-qPCR assays (Supplementary Table S1). For reasons of data evaluation, the ct-values were stratified into three different groups: a ct-value that exceeded 25 was considered as “low”, whereas a ct-value between 15 and 25 was regarded as “high”, and a ct-value below 15 was considered as a “very high” viral load.

All participating laboratories tested their own selected samples with the different LFDs by strictly following the manufacturers’ instructions. Briefly, a cotton swab was inserted into a 10% brain tissue suspension until saturated and then placed into the buffer solution where it was thoroughly mixed for about ten seconds. Between two and four drops of the buffer solution were then added to the sample pad using the disposable pipette. The reading was done 10 to 15 min afterwards, as recommended by the manufacturers. The test lines on the strips were classified by using a binomial plus/minus scoring system representing either a positive result when a red stripe appeared in the test line “T“ or a negative outcome when the test line was not visible. The test was considered valid by the appearance of a red line on the control area “C“ (Supplementary Figure S1). Also, non-infected brain homogenates (N = 20) were included (Supplementary Table S1).

### 2.4. Identification of the Binding Target of Antibodies Used in LFDs

For determining the binding target of the antibodies used on the test strips, HEK293T cells were transfected at FLI in six-well plates with 6 µg expression plasmid pCAGGS coding for the four RABV genes, N, P, M, and G, as described before [34,35]. After 24 h, the cells were suspended in 500 µl PBS. Samples from two wells were mixed and the cells were subsequently pelleted by 5 min centrifugation (1000× *g*; room temperature). The pellets were resuspended in 200 µL PBS buffer and vortexed. Eventually, 15 µL of the cell suspension were added to the buffer of the test kit and the test was evaluated as described before. This analysis was performed in duplicate per plasmid per test kit.

## 3. Results

### 3.1. Diagnostic Sensitivity and Specificity of Five LFD Test Kits

**Figure 1:**
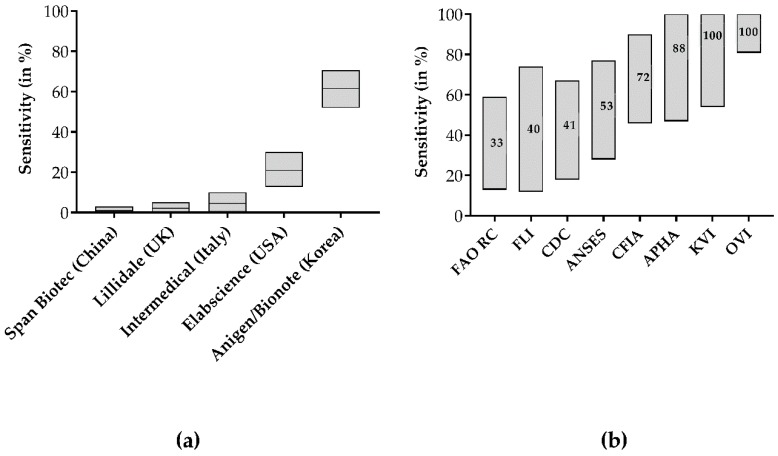
Overall sensitivities of tested different LFDs for confirmed positive samples (**a**), and the sensitivities for the Anigen/Bionote test at the different laboratories (**b**). The confidence limits are indicated as shaded boxes.

None of the negative samples tested positive in any test; thus all tests demonstrated a specificity of 100%. Test sensitivity was highly variable, ranging between 0% and 62% (Figure 1a,b, Table 1), depending on the type of LFDs. Specifically, the Span Biotech kit detected none of 105 RABV positive samples, resulting in a sensitivity of 0%. With values ranging between 1% and 3%, the Lillidale and Intermedical test kits exhibited similarly low sensitivities, whereas the Elabscience kit showed a moderately higher sensitivity of 20% (95% CI: 12.8% to 30.1%, Table 1).

The overall sensitivity of the Anigen/Bionote test for RABV was 62% (95% CI: 51.9% to 70.6%). For further analyses concerning the test agreement with DFA and RT-qPCR, only the results of Anigen/Bionote were taken into account as the sensitivities of the other four LFDs were too low to draw any further conclusions.

### 3.2. Anigen/Bionote in Interlaboratory Comparison and Agreement with DFA and RT-qPCR

**Figure 2:**
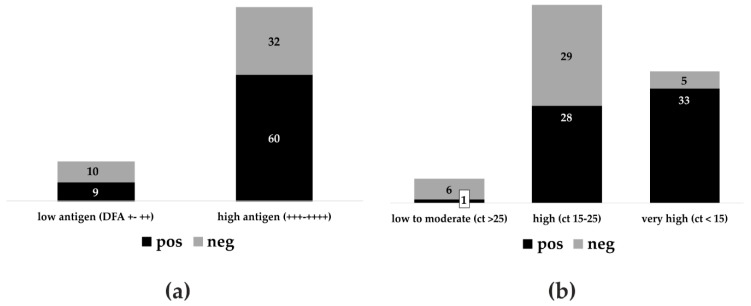
Diagnostic performance of the Anigen/Bionote LFD in relation to the antigen content as measured by DFA (**a**) and the relative viral RNA content as measured by RT-qPCR (**b**). Results are shown in absolute numbers.

When applying a post hoc stratification for factors (covariates), the Anigen/Bionote LFD test kit demonstrated a variable sensitivity between 33% and 100% in different participating laboratories (Figure 1a,b). While the sensitivity was 100% in the panels tested at KVI in Israel and OVI in South Africa, in other laboratories, much lower sensitivities were observed (Figure 1b). Specimens with a high antigen load (3+ and 4+) as measured by DFA were more likely to also test positive with the Anigen/Bionote tests than the ones with a low antigen load (+ and 2+, Figure 2a,b). The difference in the resulting sensitivities for the antigen content was not statistically significant (Fischer’s exact test, *p* = 0.31). Similarly, the sensitivity of the Anigen/Bionote test was highest (87%) in samples containing very high viral RNA loads (ct-value <15), while it decreased in samples with less RNA content (49% of ct-value 15–25, 17% of ct-value >25). Additionally, non-RABV lyssavirus positive samples, i.e., EBLV-1, EBLV-2, LLBV, and BBLV, were included among samples at APHA. All except for LLBV were detected as positive only by the Anigen/Bionote test.

### 3.3. Identification of the Binding Target of Antibodies Used in LFDs

When testing the various LFDs with different viral proteins, Span Biotech and Lillidale showed no reaction at all, whereas Intermedical and Elabscience tested positive when G-gene transfected cells were used, while Anigen/Bionote reacted specifically with N-gene transfected cells.

Of note, in the test zone “T” of Anigen/Bionote, a strong red line was clearly visible, while on the Intermedical test strips, the test line was barely visible. Also for Elabscience, where two different batches were used, a marked difference in the visibility and intensity of the test line was observed.

## 4. Discussion

In this study, different LFDs for rabies diagnosis were evaluated in regard to their diagnostic sensitivity and their agreement with DFA and RT-qPCR in order to ascertain their suitability as point-of-care diagnostics in routine surveillance. Historically, DFA was regarded as the gold standard; however, with recent updates to recommend tests by both OIE and WHO, both DFA and RT-qPCR approaches can be used as a primary diagnostic test for rabies since both demonstrate a very high (>95%) diagnostic sensitivity and specificity [8].

With sensitivities of the LFDs ranging between 0% and 62%, the outcome of our investigation confirms previous comparative analysis where different LFDs, including the Anigen/Bionote kit, showed unsatisfactory results. Although in that study the Anigen/Bionote performed the best, sub-optimal performance was still observed [30].

Apart from the Anigen/Bionote test kit, the results of the other four test kits did not differ much between the different laboratories since there were only three detections among all samples. Lillidale, Span Biotech, and Intermedical consistently failed in every laboratory. In contrast, Anigen/Biotech showed a wide range of sensitivities between 33% and 100% (Figure 1a,b, Table 1). This is perplexing, and may partly reflect the differences of our study with other published data where sensitivities of the Anigen/Bionote LFD test kit ranged between 91% and 100% [19,20,21,22,23,24,25,26,27,28,29].

With the explicit aim to include a broad diversity of RABV isolates, with respect to geographical origin, host species, and genetic background of RABV, we included different genetic lineages of all major genetic clusters [36] from most parts of rabies endemic areas. Unfortunately, none of these parameters provided any correlation to the outcome of the test result. For instance, members of one specific genetic lineage tested both positive and negative with the Anigen/Bionote test. In principle, LFD tests are based on antibody recognition of the target analyte, in our case, the lyssavirus antigen, and the performance of the test is linked to the specific characteristics of the binding antibody. No information is available on the target antigen by the manufacturers except for Anigen/Bionote [28], and our analyses using transfected cells demonstrated that only one kit recognized N protein as a target, whereas two kits detected G-protein. Because of the conserved structure of the nucleoprotein and the abundance in clinical specimens, antibodies targeting this protein are generally used for diagnostic purposes, i.e., for the DFA and the DRIT [9,11]. Conversely, it is much more difficult to verify the presence of G-protein in the brain of infected animals. Therefore, G antigen is not considered a sufficiently sensitive target for detection. In fact, this may be one reason why only the N-targeting Anigen/Bionote showed the highest sensitivities as opposed to all other tests (Figure 1a, Table 1). The absence of any reaction with transfected cells in the Span Biotech and Lillidale kits is striking and correlates to their absolute failure in detecting rabies.

Only the Anigen/Bionote tests were able to also detect three other non-RABV lyssavirus positive samples, i.e., EBLV-1, EBLV-2, and BBLV. Previous studies had also shown that the reactivity of this test is not limited to RABV, with the detection of both Phylogroup I [23,27] and Phylogroup II [21] viruses noted. With this broad reactivity, it is unlikely that the genetic background of the analyte plays an important role in modifying the test performance of the Anigen/Bionote. Parameters that influenced the likelihood of test agreement with the established DFA and RT-qPCR methods were a high antigen as well as high viral RNA content, indicating an effect of disease progression on the test performance, which would be a severe limitation of these tests. Such effects of different populations on test characteristics are often seen in validation studies [37].

Still, the lack of test agreement with the DFA and RT-qPCR remains puzzling against the background that most of the samples used for the interlaboratory comparison had already been confirmed highly positive in DFA.

For reasons that also remain unknown, fresh samples from the field, used at KVI in Israel and OVI in South Africa, tended to yield better agreements with standard methods than archived samples, even though these were tested positive in RT-qPCR and DFA after years of storage. This phenomenon was observed previously with laboratory [30] and field investigations [19]. The latter study also demonstrated an increased sensitivity when modifying the manufacturer’s instruction and eliminating the first dilution step, an effect also observed under laboratory conditions [30]. In fact, the sensitivity was also increased when the test protocol by Lechenne et al. 2016, whereby the initial dilution step is omitted, was additionally applied at the FAO reference centre in the present study (Supplementary Table S1). Together these data suggest that a modification of the manufacturer’s instructions may increase sensitivity of the Anigen/Bionote test kit.

Another inter-laboratory comparison with two LFDs, including the Anigen/Bionote, was recently performed using a panel of ten anonymized samples of experimentally infected mouse brains [20]. For a single lot of Anigen/Bionote test kits, an overall concordance of results of 100% was achieved amongst the participating laboratories, and in comparison to the DFA, for RABV-infected tissue. However, these results must be interpreted carefully regarding the diagnostic sensitivity of the test as the sample size was limited, and did not necessarily reflect the variation in antigen load that would be observed in a diagnostic laboratory. Furthermore, the sample dilution used was lower than that found in the manufacturer’s instructions. In the same laboratory (ANSES), the sensitivity of the Anigen/Bionote kit in the present study was below average (Figure 1b), supporting previous concerns about batch-to-batch variation in the quality of those tests [30], and a trend towards reduced sensitivity on samples with lower antigen content, as demonstrated here.

In the context of shortages in rabies diagnostics in low resource settings, the development of rapid, reasonably priced, and user-friendly solutions to detect rabies virus in brain material could be a major step towards disease control and elimination. However, high quality is crucial as false-negative test results would not only promote negligence in a global epidemiological context but may discourage exposed individuals from seeking prompt medical care including PEP, putting them at an important risk of fatal outcome. Additionally, even though the specificity was high in this and other studies, false positive outcomes are also unacceptable as they lead to an incorrect distribution of expensive and limited medical resources and biologics.

With the increasing demand for point of care diagnostics, a growing but non-transparent landscape of commercially available LFDs for rabies diagnosis has emerged. It is not clear how many of the approximately 15 LFD test kits which are currently available online are actually sold under different brand names while they may originate from only a few manufacturers. Furthermore, none of the manufacturers provided detailed insight into the principles on which their tests are based, i.e., the specific antibodies they use on the test strip.

This lack of transparency, proper validation, and thus reliability limits the use of LFDs and the results of our study advocate for stricter quality controls and approval procedures, especially when taking the international goal to reach zero human deaths due to dog-mediated rabies by 2030 into account. In some countries, for example in Germany, where diagnostics for notifiable animal diseases need to obtain marketing authorization, such tests would not fulfill the respective criteria.

There are clear guidelines for the development and validation of tests for veterinary diagnostics. Following these guidelines, a change of procedures after licensure, as it has been done in some field studies (e.g., [19]), would not be acceptable [38]. Another major component that had been addressed before [19,30] is the manufacturers’ instructions, which should be more specific for sampling and sample preparation, and secondly, concern the use of saliva instead of brain material. Except for Anigen/Bionote, all other tests still mention saliva as an analyte, which is likely to give false negative results because of intermittent shedding of virus in saliva or limited amount of virus in saliva below the limit of detection [39]. The declaration of some manufacturers that the purpose of the kit is “for research only” does not absolve them from their responsibilities in offering a test with an appropriate quality as those tests are most likely bought by customers other than research scientists. In response to the results obtained, all manufacturers were contacted but the response received was limited to no responses at all. Intermedical, Elabscience, and Lillidale sent new batches for re-testing and after confirming the limited quality, at least Lillidale guaranteed to stop sales.

## 5. Conclusions

The increasing use of LFDs as a basis for surveillance, disease control, and medical intervention [19] indicates the need to overcome resource- and operational limitations in current rabies diagnosis. Nevertheless, massive improvements need to happen before tests, even the Anigen/Bionote test, can be unconditionally recommended by the OIE. Simply encouraging the producers to substantially improve and assure the quality of their test kits, as done by the previous study [30], did not show any significant development or changes. Therefore, the quality and evaluation of those tests should be controlled through standardized approval procedures following OIE recommendations [38]. In order to increase the pressure on manufacturers, for instance, only those kits that have passed such licensure should be used in the frame of UAR-supported rabies control programs.

## Figures and Tables

**Table 1:** Summary of results for the different LFD tests for rabies positive and negative samples.

Manufacturer	RABV Pos		RABV Neg		Sensitivity	95% CI
	LFD Pos	LFD Neg	LFD Pos	LFD Neg		
Span Biotec (China)	0	105	0	12	0%	0% to 3%
Lillidale (UK)	1	103	0	12	1%	0.2% to 5%
Intermedical (Italy)	2	66	0	11	3%	0.3% to 10%
Elabscience (China/USA)	19	74	0	12	20%	12.8% to 30.1%
Anigen/Bionote (Korea)	69	43	0	16	62%	51.9% to 70.6%

## Supplementary Materials

The following are available online at https://www.mdpi.com/2414-6366/5/1/13/s1, Figure S1: positive direct fluorescent antibody test (DFA, a), positive direct rapid immunochemical test (DRIT, b) and negative LFD result (Anigen/Bionote, c) of a rabid fox (A17-3454 AZ) infected with the South Central Skunk RABV variant, a and b: magnification = 200×; Table S1: Test results and details of samples tested.

## References

[1] Hampson K., Coudeville L., Lembo T., Sambo M., Kieffer A., Attlan M., Barrat J., Blanton J.D., Briggs D.J., Cleaveland S. (2015). Estimating the global burden of endemic canine rabies. PLOS Negl. Trop. Dis..

[2] World Health Organization (2018). WHO expert consultation on rabies, third report. World Health Organ. Tech. Rep. Ser..

[3] Fooks A.R., Cliquet F., Finke S., Freuling C., Hemachudha T., Mani R.S., Müller T., Nadin-Davis S., Picard-Meyer E., Wilde H. (2017). Rabies. Nat. Rev. Dis. Primers..

[4] Amarasinghe G.K., Arechiga Ceballos N.G., Banyard A.C., Basler C.F., Bavari S., Bennett A.J., Blasdell K.R., Briese T., Bukreyev A., Cai Y. (2018). Taxonomy of the order Mononegavirales: Update 2018. Arch. Virol..

[5] Finke S., Conzelmann K.K. (2005). Replication strategies of rabies virus. Virus Res..

[6] Abela-Ridder B. (2017). Rabies elimination: Protecting vulnerable communities through their dogs-Authors‘ reply. Lancet Glob. Health.

[7] World Health Organization, Food and Agriculture Organization of the United Nations, World Organisation for Animal Health (2018). Global Alliance for Rabies Control. Zero by 30: The Global Strategic Plan to Prevent Human Deaths from Dog-Transmitted Rabies by 2030 In Executive Summary.

[8] Office International des Epizooties (OIE) (2018). Chapter 2.1.17. Rabies (Infection with Rabies virus and other Lyssaviruses). OIE Manual of Diagnostic Tests and Vaccines for Terrestrial Animals.

[9] Rupprech C.E., Fooks A.R., Abela-Ridder B., WHO (2018). Chapter 11. The direct florescent antibody test (DFAT). Laboratory Techniques in Rabies.

[10] Lembo T., Niezgoda M., Velasco-Villa A., Cleaveland S., Ernest E., Rupprecht C.E. (2006). Evaluation of a direct, rapid immunohistochemical test for rabies diagnosis. Emerg. Infect. Dis..

[11] Rupprech C.E., Fooks A.R., Abela-Ridder B., World Health Organization (2018). Chapter 12. The direct rapid immunohistochemistry test (DRIT) for the detection of lyssavirus antigens. Laboratory Techniques in Rabies.

[12] Rupprech C.E., Fooks A.R., Abela-Ridder B., World Health Organization (2018). Chapter 9. Virus isolation in cell culture: The rabies tissue culture infection test (RTCIT). Laboratory Techniques in Rabies.

[13] Rupprech C.E., Fooks A.R., Abela-Ridder B., World Health Organization (2018). Chapter 8. Virus isolation in animals: The mouse inoculation test (MIT). Laboratory Techniques in Rabies.

[14] Banyard A.C., Horton D., Freuling C., Müller T., Fooks A.R. (2013). Control and prevention of canine rabies: The need for building laboratory-based surveillance capacity. Antivir. Res..

[15] Cleaveland S., Beyer H., Hampson K., Haydon D., Lankester F., Lembo T., Meslin F.X., Morters M., Mtema Z., Sambo M. (2014). The changing landscape of rabies epidemiology and control. Onderstepoort J. Vet. Res..

[16] Bourhy H., Dautry-Varsat A., Hotez P.J., Salomon J. (2010). Rabies, Still Neglected after 125 Years of Vaccination. PLOS Negl. Trop. Dis..

[17] O’Farrell B. (2015). Lateral Flow Technology for Field-Based Applications-Basics and Advanced Developments. Top. Companion Anim. Med..

[18] Rupprech C.E., Fooks A.R., Abela-Ridder B., World Health Organization (2018). Chapter 17. Rapid immunochromatographic tests for the detection of rabies virus antigens in brain material. Laboratory Techniques in Rabies.

[19] Léchenne M., Naïssengar K., Lepelletier A., Alfaroukh I.O., Bourhy H., Zinsstag J., Dacheux L. (2016). Validation of a Rapid Rabies Diagnostic Tool for Field Surveillance in Developing Countries. PLOS Negl. Trop. Dis..

[20] Servat A., Robardet E., Cliquet F. (2019). An inter-laboratory comparison to evaluate the technical performance of rabies diagnosis lateral flow assays. J. Virol. Methods.

[21] Markotter W., York D., Sabeta C.T., Shumba W., Zulu G., Roux Le K., Nel L.H. (2009). Evaluation of a rapid immunodiagnostic test kit for detection of African lyssaviruses from brain material. Onderstepoort J. Vet. Res..

[22] Reta T., Teshale S., Deresa A., Ali A., Getahun G., Baumann M.P.O., Müller T., Freuling C.M. (2013). Evaluation of Rapid Immunodiagnostic Test for Rabies Diagnosis Using Clinical Brain Samples in Ethiopia. J. Vet. Sci. Med. Diagn..

[23] Servat A., Picard-Meyer E., Robardet E., Muzniece Z., Must K., Cliquet F. (2012). Evaluation of a Rapid Immunochromatographic Diagnostic Test for the detection of rabies from brain material of European mammals. Biologicals.

[24] Voehl K.M., Saturday G.A. (2014). Evaluation of a rapid immunodiagnostic rabies field surveillance test on samples collected from military operations in Africa, Europe, and the Middle East. US Army Med. Dep. J..

[25] Pranoti S., Singh C.K., Deepti N. (2015). Comparison of immunochromatographic diagnostic test with heminested reverse transcriptase polymerase chain reaction for detection of rabies virus from brain samples of various species. Vet. World.

[26] Yang D.K., Shin E.K., Oh Y.I., Lee K.W., Lee C.S., Kim S.Y., Lee J.A., Song J.Y. (2012). Comparison of four diagnostic methods for detecting rabies viruses circulating in Korea. J. Vet. Sci..

[27] Certoma A., Lunt R.A., Vosloo W., Smith I., Colling A., Williams D.T., Tran T., Blacksell S.D. (2018). Assessment of a Rabies Virus Rapid Diagnostic Test for the Detection of Australian Bat Lyssavirus. Trop. Med. Infect. Dis..

[28] Kang B., Oh J., Lee C., Park B.K., Park Y., Hong K., Lee K., Cho B., Song D. (2007). Evaluation of a rapid immunodiagnostic test kit for rabies virus. J. Virol. Methods.

[29] Nishizono A., Khawplod P., Ahmed K., Goto K., Shiota S., Mifune K., Yasui T., Takayama K., Kobayashi Y., Mannen K. (2008). A simple and rapid immunochromatographic test kit for rabies diagnosis. Microbiol. Immunol..

[30] Eggerbauer E., de Benedictis P., Hoffmann B., Mettenleiter T.C., Schlottau K., Ngoepe E.C., Sabeta C.T., Freuling C.M., Müller T. (2016). Evaluation of Six Commercially Available Rapid Immunochromatographic Tests for the Diagnosis of Rabies in Brain Material. PLOS Negl. Trop. Dis..

[31] Gay C.G., Morgan A.P. (1993). Licensing veterinary diagnostic test kits in the United States. Clin. Immunol. Newslett..

[32] Anonym (2013). Animal Health Act (TierGesG). Federal Law Gazette.

[33] Clopper C.J., Pearson E.S. (1934). The Use of Confidence or Fiducial Limits Illustrated in the Case of the Binomial. Biometrika.

[34] Conzelmann K.H., Cox J.H., Schneider L.G., Thiel H.J. (1990). Molecular Cloning and Complete Nucleotide Sequence of the Attenuated Rabies Virus SAD B19. Virology.

[35] Finke S., Granzow H., Hurst J., Pollin R., Mettenleiter T.C. (2010). Intergenotypic replacement of lyssavirus matrix proteins demonstrates the role of lyssavirus M proteins in intracellular virus accumulation. J. Virol..

[36] Fischer S., Freuling C.M., Muller T., Pfaff F., Bodenhofer U., Hoper D., Fischer M., Marston D.A., Fooks A.R., Mettenleiter T.C. (2018). Defining objective clusters for rabies virus sequences using affinity propagation clustering. PLOS Negl. Trop. Dis..

[37] Greiner M., Gardner I.A. (2000). Epidemiologic issues in the validation of veterinary diagnostic tests. Prev. Vet. Med..

[38] Office International des Epizooties (OIE) (2018). Chapter 1.1.6 Principles and methods of validation of diagnostic assays for infectious diseases. OIE Manual of Diagnostic Tests and Vaccines for Terrestrial Animals.

[39] Hanlon C., Jackson A.C. (2013). Rabies in Terrestrial Animals. Rabies: Scientific Basis of the Disease and Its Management.

