# Supplementary material for: Further Evidence of Inadequate Quality in Lateral Flow Devices Commercially Offered for the Diagnosis of Rabies
Source: Trop Med Infect Dis. 2020 Jan 18;5(1):13. doi: 10.3390/tropicalmed5010013 (PMC7157750; doi:10.3390/tropicalmed5010013)
Supplement: Supplementary file 1 [file tropicalmed-05-00013-s001.zip › Supplementary Table 1.pdf]

Lab	LabnegID	host	virus	lineage	origin	DFA	qRTnegPCRnegResult	Viral load	InterMedical	Anigen/Bionote	Anigen/Bionote modified	Elabscience	Rabies Ag (Span Biotech)	Lillidale
ANSES	DR-1793	cat	-	-	Luxembourg	neg	no Ct		neg	neg		not tested	neg	neg
ANSES	DR-1788	fox	-	-	Luxembourg	neg	no Ct		neg	neg		not tested	neg	neg
ANSES	DR-1784	fox	-	-	Luxembourg	neg	no Ct		neg	neg		neg	neg	neg
ANSES	GS-5 lot 05-18 (1/50)	fox ® mouse	RABV	Cosmopolitan lineage-WE	France	2	19,70	high (15-25)	neg	neg		neg	neg	neg
ANSES	GS-5 lot 03-18 (1/30)	fox ® mouse	RABV	Cosmopolitan lineage-WE	France	3	18,90	high (15-25)	neg	neg		neg	neg	neg
ANSES	GS-7 lot 14-18	fox ® mouse	RABV	Cosmopolitan lineage-WE	France	3	10	very high (5-15)	neg	pos		(pos) Doubtful	neg	neg
ANSES	RABV Maroc lot 12-17	dog ® mouse	RABV	Cosmopolitan-Africa-1	Morocco	3	17,50	high (15-25)	neg	pos		neg	neg	neg
ANSES	Slovaquie lot 10-16	fox ® mouse	RABV	Cosmopolitan lineage-NEE	Slovakia	3	18,80	high (15-25)	neg	pos		neg	neg	neg
ANSES	DR-1778	reindeer	RABV	Arctic Fox RV	Norway	3	28,20	low	neg	neg		neg	neg	neg
ANSES	DR-0914	fox	RABV	Cosmopolitan lineage-EE	Albania	1	16,40	high (15-25)	neg	pos		neg	neg	neg
ANSES	DR-1068	fox	RABV	Vaccine strain	Hungaria	1	25,60	low	neg	neg		neg	neg	neg
ANSES	DR-1037	cattle	RABV	Vaccine strain	Romania	2	14,50	very high (5-15)	neg	pos		neg	neg	neg
ANSES	DR-1333	fox	RABV	Cosmopolitan lineage-NEE	Romania	1	16,80	high (15-25)	neg	neg		neg	neg	neg
ANSES	DR-1335	fox	RABV	Cosmopolitan lineage-NEE	Romania	2	16,40	high (15-25)	neg	neg		neg	neg	neg
ANSES	DR-1343	cat	RABV	Cosmopolitan lineage-NEE	Moldova	2	17,30	high (15-25)	neg	neg		neg	neg	neg
ANSES	DR-1790	fox	RABV	Cosmopolitan lineage-C	Lituania	3	11,40	very high (5-15)	neg	pos		neg	neg	neg
ANSES	DR-0641	dog	RABV	Cosmopolitan lineage-EE	Greece	1	19,60	high (15-25)	neg	pos		neg	neg	neg
ANSES	DR-0627	fox	RABV	Cosmopolitan lineage-EE	Greece	1	18,20	high (15-25)	neg	pos		neg	neg	neg
ANSES	DR-0653	fox	RABV	Cosmopolitan lineage-EE	Greece	2	19,10	high (15-25)	neg	pos		neg	neg	neg
ANSES	DR-0654	fox	RABV	Cosmopolitan lineage-EE	Greece	1	18,60	high (15-25)	neg	neg		neg	neg	neg
Italy	10VIR/5493-15	WILDCAT	RABV	AFRICA 3	BOTSWANA	4	17,20	high (15-25)	not tested	pos	pos	not tested	neg	neg
Italy	10VIR/5493-8	DOG	RABV	COSMOPOLITAN (EX-AFRICA 1)	BOTSWANA	4	19,16	high (15-25)	not tested	pos	pos	not tested	neg	neg
Italy	10VIR/5493-10	BOVINE	RABV	COSMOPOLITAN (EX-AFRICA 1)	BOTSWANA	4	14,30	very high (5-15)	not tested	pos	pos	not tested	neg	neg
Italy	RS13/266 -4	BOVINE	RABV	COSMOPOLITAN (EX-AFRICA 1)	UGANDA	4	20,66	high (15-25)	not tested	neg	pos	not tested	neg	neg
Italy	RS13/266 -5	DOG	RABV	COSMOPOLITAN (EX-AFRICA 1)	UGANDA	4	16,65	high (15-25)	not tested	neg	pos	not tested	neg	neg
Italy	RS13/266 -12	DOG	RABV	COSMOPOLITAN (EX-AFRICA 1)	UGANDA	4	20,32	high (15-25)	not tested	neg	pos	not tested	neg	neg
Italy	RS13/116-8	BOVINE	RABV	American-Indigenous (vampire bat)	Brazil	4	22,28	high (15-25)	not tested	neg	pos	not tested	neg	neg
Italy	RS13/116-17	BOVINE	RABV	American-Indigenous (vampire bat)	Brazil	4	22,14	high (15-25)	not tested	neg	pos	not tested	neg	neg
Italy	RS13/116-38	BOVINE	RABV	American-Indigenous (vampire bat)	Brazil	4	23,56	high (15-25)	not tested	neg	pos	not tested	neg	neg
Italy	RS13/1249	mice replicated (ex-BAT)	RABV	American-Indigenous (insectivorous bat)	Brazil	4	23,19	high (15-25)	not tested	neg	pos	not tested	neg	neg
Italy	RS11/3609-5	POTOS FLAVUS	RABV	American-Indigenous (insectivorous bat)	Brazil	4	not tested		not tested	pos	pos	not tested	neg	neg
Italy	10RD/2322	FOX	RABV	Italy 1	Italy	4	not tested		not tested	neg	neg	not tested	neg	neg
Italy	10RD/1450	FOX	RABV	Italy 2	Italy	4	22,82	high (15-25)	not tested	neg	pos	not tested	neg	neg
Italy	10RD/1000	FOX	RABV	Italy 2a	Italy	4	16,50	high (15-25)	not tested	neg	pos	not tested	neg	neg
Italy	07VIR/6235- 3	DOG?	RABV	AFRICA 2	Mauritania	4	not tested		not tested	pos	pos	not tested	neg	neg
Italy	07VIR/6097 (253)	DOG?	RABV	AFRICA 2	Niger	4	not tested		not tested	neg	neg	not tested	neg	neg
Italy	18RD/666-2	DOG	RABV	AFRICA 2	Liberia	4	not tested		not tested	neg	pos	not tested	neg	neg
Italy	18RD/666-4	DOG	RABV	AFRICA 2	Liberia	4	not tested		not tested	pos	pos	not tested	neg	neg
Italy	18RD/666-1	DOG			Liberia	neg	no cT		not tested	neg	neg	not tested	neg	neg
Italy	RS13/266 -11	DOG			UGANDA	neg	not tested		not tested	neg	neg	not tested	neg	neg
KVI Israel	336706	Jackal	RABV	Cosmopolitan (Middle East)	Israel	4	11,90	very high (5-15)	neg	pos		pos	not tested	not tested
KVI Israel	320155	Dog	RABV	Cosmopolitan (Middle East)	Israel	4	12,30	very high (5-15)	neg	pos		neg	not tested	not tested
KVI Israel	315175	Cattle	RABV	Cosmopolitan (Middle East)	Israel	4	9,10	very high (5-15)	neg	pos		pos	not tested	not tested
KVI Israel	318829	Sheep	RABV	Cosmopolitan (Middle East)	Israel	4	10,50	very high (5-15)	neg	pos		pos	not tested	not tested
KVI Israel	305400	Cat	RABV	Cosmopolitan (Middle East)	Israel	4	12,90	very high (5-15)	neg	pos		neg	not tested	not tested
KVI Israel	317604	Badger	RABV	Cosmopolitan (Middle East)	Israel	4	11,10	very high (5-15)	neg	pos		pos	not tested	not tested
KVI Israel	337355	Jackal	-	-		neg	no cT		neg	neg		neg	not tested	not tested
KVI Israel	337447	Cat	-	-		neg	no cT		neg	neg		neg	not tested	not tested
KVI Israel	337443	Dog	-	-		neg	no cT		neg	neg		neg	not tested	not tested
KVI Israel	337217	Cattle	-	-		neg	no cT		neg	neg		neg	not tested	not tested
CDC	A19-0514	Sheep	NA	NA	Georgia, USA	neg	no cT		invalid test	neg		neg	neg	neg
CDC	A19-0070	Raccoon	RABV	Eastern Raccoon RV	Tennessee, USA	4	20,88	high (15-25)	neg	pos (weak)		neg	neg	neg
CDC	A19-0069	Skunk	RABV	North Central Skunk RV	Tennessee, USA	4	21,77	high (15-25)	neg	neg		neg	neg	neg
CDC	A19-0484	Dog	RABV	Cosmolitan Dog (AF-4) RV	Egypt (translocated to KS USA)	4	26,04	low	neg	neg		neg	neg	neg
CDC	A18-1142	Bovine	RABV	Arizona Gray Fox RV	Arizona, USA	4	22,81	high (15-25)	neg	neg		neg	neg	neg
CDC	A18-1690	Dog	RABV	Tadarida brasiliensis (Tb) RV	Georgia, USA	4	21,97	high (15-25)	neg	pos (weak)		neg	neg	neg
CDC	A18-1274	Goat	RABV	North Central Skunk RV	North Dakota, USA	4	23,10	high (15-25)	neg	neg		neg	neg	neg
CDC	A18-0238	Cat	RABV	Lasiurus cinereus RV	North Carolina, USA	4	25,37	low	invalid test	neg		neg	neg	neg
CDC	A18-1123	Dog	RABV	Eastern Raccoon RV	Delaware, USA	4	22,99	high (15-25)	neg	neg		neg	neg	neg
CDC	A17-3454	Fox	RABV	South Central Skunk RV	Arizona, USA	4	20,28	high (15-25)	invalid test	neg		neg	neg	neg
CDC	A19-0072	Raccoon	NA	NA	Kentucky, USA	neg	no cT		neg	neg		neg	neg	neg
CDC	A18-1145	Dog	NA	NA	Virgin Islands (US Territory)	neg	no cT		neg	neg		neg	neg	neg
CDC	A18-2091	Raccoon	RABV	Eastern Raccoon RV	Georgia, USA	4	20,32	high (15-25)	neg	pos (weak)		neg	neg	neg
CDC	A18-3256	Fox	RABV	Arctic Fox RV	Alaska, USA	4	23,25	high (15-25)	neg	pos (weak)		neg	neg	neg
CDC	A17-4048	Skunk	RABV	Western Eptesicus fuscus RV	Colorado, USA	4	21,98	high (15-25)	neg	neg		neg	neg	neg
CDC	A17-4100	Fox	RABV	Arizona Gray Fox RV	Arizona, USA	4	24,60	high (15-25)	neg	neg		neg	neg	neg
CDC	A17-4241	Dog	RABV	Cosmolitan Dog (AF-4) RV	Egypt (translocated to CT USA)	4	22,93	high (15-25)	neg	pos (weak)		neg	neg	neg
CDC	A17-3417	Fox	RABV	South Central Skunk RV	New Mexico, USA	4	19,31	high (15-25)	neg	pos (weak)		neg	neg	neg
CDC	A15-2820 (15-3409)	Gray Fox	RABV	Myotis sp RV	Oregon, USA	4	25,62	low	invalid test	(pos) Doubtful		neg	neg	neg
CDC	A15-2815 (13V01325)	Fox	RABV	Myotis sp RV	Oregon, USA	4	26,33	low	neg	neg		neg	neg	neg
OVI	001/19	Bovine	RABV	Mongoose variant	Clocolan, Free State, SA	3	23,72	high (15-25)	neg	pos		neg	neg	neg
OVI	022/19	Mongoose	RABV	Mongoose variant	Coligny, North West, SA	4	13,41	very high (5-15)	neg	pos		pos	neg	neg
OVI	044/19	Jackal	RABV	canid variant	Knopfontein, North West, SA	4	24,17	high (15-25)	pos	pos		pos	neg	neg
OVI	045/19	Dog	RABV	canid variant	Jappi, Limpopo, SA	4	15,05	high (15-25)	neg	pos		neg	neg	neg
OVI	049/19	Jackal	RABV	canid variant	Naledi, North West, SA	4	12,85	very high (5-15)	pos	pos		neg	neg	neg
OVI	065/19	Dog	RABV	canid variant	Polokwane, Limpopo, SA	4	10,82	very high (5-15)	neg	pos		neg	neg	neg
OVI	076/19	Bovine	RABV	canid variant	Ganyesa, North West, SA	4	20,71	high (15-25)	neg	pos		neg*	neg	neg
OVI	081/19	Meerkat	RABV	Mongoose variant	Ladybrand, Free State, SA	4	13,16	very high (5-15)	neg	pos		neg	neg	neg
OVI	384/18	Dog	RABV	canid variant	Nkomazi, Mpumalanga, SA	4	24,48	high (15-25)	neg	pos		neg	neg	neg
OVI	391/18	Dog	RABV	canid variant	Klerksdorp, North West, SA	4	19,51	high (15-25)	neg	pos		neg	neg	neg
OVI	396/18	Rhino	RABV	canid variant	Windhoek, Namibia	4	13,20	very high (5-15)	neg	pos		neg	neg	neg
OVI	505/18	Dog	RABV	canid variant	Schwezernaicker, NW, SA	4	12,11	very high (5-15)	neg	pos		neg	neg	neg
OVI	541/18	Bat Eared fox	RABV	canid variant	Kuruman, Northern Cape, SA	4	20,89	high (15-25)	neg	pos		neg	neg	neg
OVI	551/18	Dog	RABV	canid variant	Madidi, North West, SA	4	20,88	high (15-25)	neg	pos		neg	neg	neg
OVI	555/18	Dog	RABV	canid variant	Tzaneen, Limpopo, SA	4	19,74	high (15-25)	neg	pos		neg	neg	1
OVI	557/18	Dog	RABV	canid variant	Bushbuckridge, MP, SA	4	20,13	high (15-25)	neg	pos		neg	neg	neg
OVI	560/18	Dog	RABV	canid variant	Makhado, Limpopo, SA	4	25,94	low	neg	pos		neg	neg	neg
CVI						neg	no cT		not tested	neg		neg	neg	neg
CVI						neg	no cT		not tested	neg		neg	neg	neg
CVI	V113 (H/119/86/D University of Berne)	dog	RABV	Sri Lankan dog	Sri Lanka	4	7,10	very high (5-15)	not tested	+		+	neg	neg
CVI	OLF-18-00127	dog	RABV	Arctic Fox RV	Canada	1	20,10	high (15-25)	not tested	neg		neg	neg	neg
CVI	OLF-17-00722	dog	RABV	Arctic Fox RV	Canada	1	17,90	high (15-25)	not tested	neg		neg	neg	neg
CVI	OLF-17-00710	dog	RABV	Arctic Fox RV	Canada	1	18,20	high (15-25)	not tested	neg		neg	neg	neg
CVI	V120 (82 University of Berne)	dog	RABV	Nepal dog	Nepal	3	6,10	very high (5-15)	not tested	+		+	neg	neg
CVI	V050 (RV424 Central Veterinary Lab, Weybridge GB)	yellow mongoose (MBr pass)	RABV	Africa mongoose	RSA	4	7,70	very high (5-15)	not tested	+		+	neg	neg
CVI	V218 (RR4-73 Texas Department of State Health Services)	coyote (MBr pass)	RABV	Mexican dog/coyote	Mexico	4	9,30	very high (5-15)	not tested	+		+	neg	neg
CVI	OLF-18-01902	dog	RABV	Arctic Fox RV	Canada	3	9,70	very high (5-15)	not tested	+		+	neg	neg
CVI	OLF-18-01669	skunk	RABV	BBCAN1	Canada	4	11,90	very high (5-15)	not tested	+		neg	neg	neg
CVI	OLF-18-1582	little brown bat (Myotis lucifugus)	RABV	MYCAN	Canada	4	13,80	very high (5-15)	not tested	neg		neg	neg	neg
CVI	V737 (00R025 Gyeongsang National Univeristy, Korea)	raccoon dog	RABV	Korea raccoon dog	Korea	4	12,90	very high (5-15)	not tested	+		neg	neg	neg
CVI	V650 (T97-0372 California Department of Public Health)	striped skunk	RABV	California skunk	USA	3	16,50	high (15-25)	not tested	+		+	neg	neg
CVI	V903 (Zoonosis Control Center Sao Paulo Brazil 1989)	dog	RABV	BRL-1a	Brazil	2	10,60	very high (5-15)	not tested	+		+	neg	neg
CVI	V908 (Zoonosis Control Center Sao Paulo Brazil 1997)	Histiotus velatus (MBr pass)	RABV	BRL-2	Brazil	3	14,80	very high (5-15)	not tested	+		neg	neg	neg
CVI	V905 (Zoonosis Control Center Sao Paulo Brazil 1999)	horse	RABV	BRL-3	Brazil	1	18,80	high (15-25)	not tested	neg		neg	neg	neg
CVI	OLF-16-00270	raccoon	RABV	MidAtlantic Raccoon	Canada	3	9,80	very high (5-15)	not tested	+		neg	neg	neg
CVI	OLF-17-00231	raccoon	RABV	MidAtlantic Raccoon	Canada	3	12,50	very high (5-15)	not tested	+		neg	neg	neg
CVI	OLF-18-01803	striped skunk	RABV	Western skunk	USA	4	9,90	very high (5-15)	not tested	+		+	neg	neg
APHA	RV50	Bat	RABV		USA	4	20,97	high (15-25)	neg	neg		neg	neg	neg
APHA	RV61	Arc-like	RABV	Arc-like	India	3	16,17	high (15-25)	neg	+		neg	neg	neg
APHA	RV313	Fox	RABV	cosmopolitan	Germany	3	16,10	high (15-25)	Not tested	(1) Doubtful		neg	neg	neg
APHA	RV334		RABV	Vaccine strain	China	2	16,57	high (15-25)	Not Tested	pos		(1) Doubtful	neg	neg
APHA	RV410		RABV	Africa 3	RSA	4	18,73	high (15-25)	neg	pos		(1) Doubtful	neg	neg
APHA	RV437	Raccoon Dog	RABV	cosmopolitan	Estonia	3	17,65	high (15-25)	neg	(1) Doubtful		(1) Doubtful	neg	neg
APHA	RV1237	Deer	RABV	cosmopolitan	Serbia	2	18,71	high (15-25)	Not Tested	(1) Doubtful		neg	neg	neg
APHA	RV2417	Dog	RABV		Sri Lanka	2	13,34	very high (5-15)	neg	pos		neg	neg	neg
APHA	RV20	bat	EBLV-1	subtype 1a	Germany	3	17.92		neg	(1) Doubtful		neg	invalid test	neg
APHA	RV628	bat	EBLV-2		UK	2	not tested		neg	+		neg	neg	neg
APHA	RV2507	bat	BBLV		Germany	1	not tested		neg	+		neg	neg	neg
APHA	RV3208	bat	LLEBV		Spain	2	27.28		neg	neg		neg	neg	neg
APHA	NA	Mouse	-ve			neg	no cT		neg	neg		neg	neg	neg
APHA	NA	Mouse	-ve			neg	no cT		neg	neg		neg	neg	neg
FLI	13315	horse	RABV	cosmopolitan	Germany	4	13,45	very high (5-15)	n/a	neg		neg	neg	neg
FLI	5013	fox	RABV	cosmopolitan	Germany	4	15,00	very high (5-15)	neg	neg		neg	neg	neg
FLI	35005	cat	RABV	cosmopolitan	Germany	4	12,88	very high (5-15)	neg	+		+	neg	neg
FLI	5481	sheep	RABV	cosmopolitan	Germany	3	16,10	high (15-25)	neg	neg		neg	neg	neg
FLI	5465	fox	RABV	cosmopolitan	Germany	4	13,03	very high (5-15)	neg	+		neg	neg	neg
FLI	11571	fox	RABV	cosmopolitan	Germany	4	14,13	very high (5-15)	neg	neg		neg	neg	not tested
FLI	11565	fox	RABV	cosmopolitan	Germany	4	13,93	very high (5-15)	neg	+		neg	neg	neg
FLI	13638	fox	RABV	cosmopolitan	Germany	4	14,54	very high (5-15)	neg	neg		neg	neg	neg
FLI	31181	deer	RABV	cosmopolitan	Germany	3	13,41	very high (5-15)	neg	+		neg	neg	neg
FLI	31827	dog	RABV	cosmopolitan	Germany	4	18,22	high (15-25)	neg	neg		neg	not tested	not tested
FLI	5483	fox	RABV	cosmopolitan	Germany	2	23,65	high (15-25)	Not tested	not tested		not tested	neg	neg
