# Supplementary figures and images for: Further Evidence of Inadequate Quality in Lateral Flow Devices Commercially Offered for the Diagnosis of Rabies
Source: Trop Med Infect Dis. 2020 Jan 18;5(1):13. doi: 10.3390/tropicalmed5010013 (PMC7157750; doi:10.3390/tropicalmed5010013)

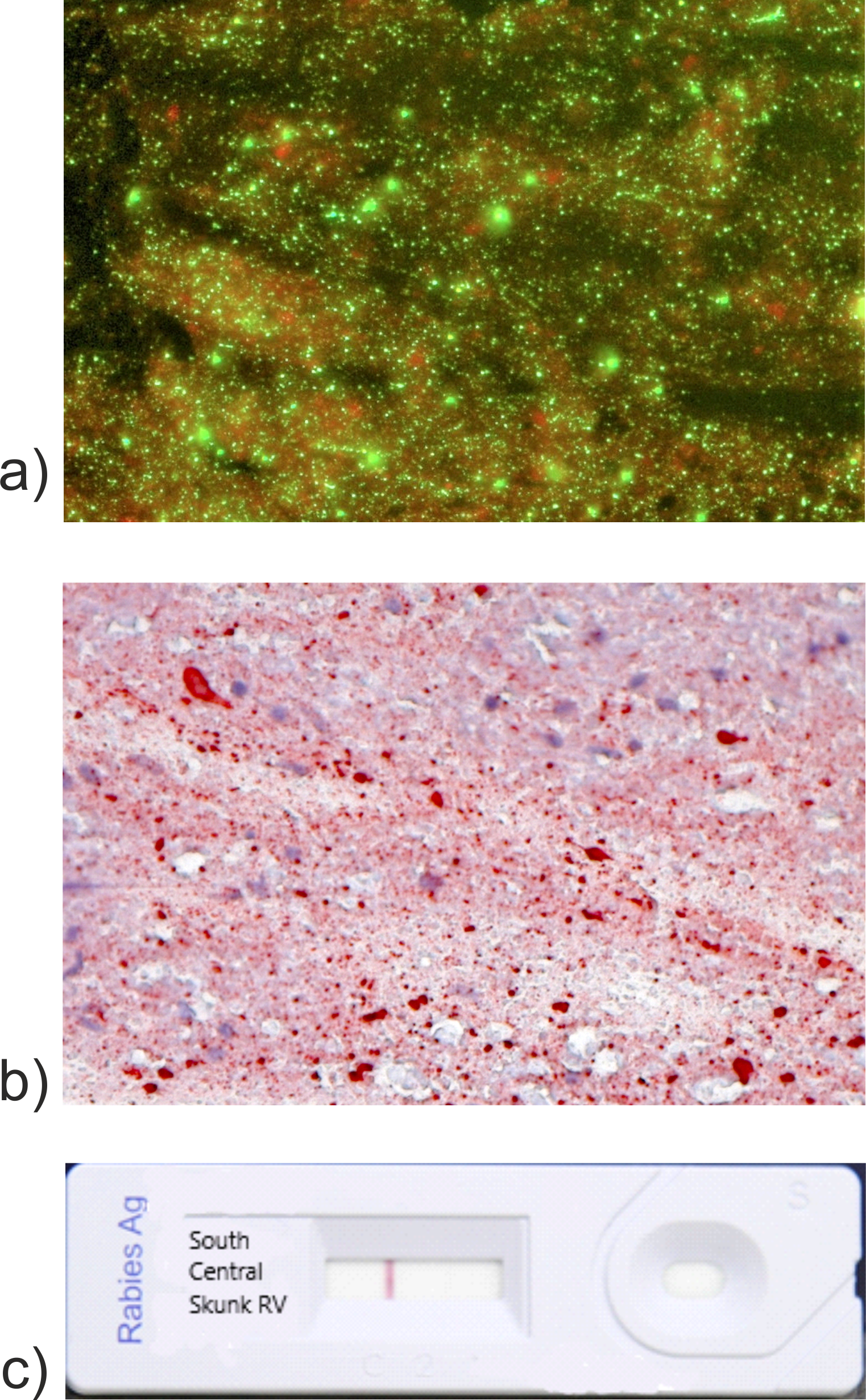

Supplement: Supplementary file 1 [file tropicalmed-05-00013-s001.zip › Supplementary_Figure.png]
